# Supplementary material for: Evaluation of Household Preparedness and Risk Factors for Cutaneous Leishmaniasis (CL) Using the Community Assessment for Public Health Emergency Response (CASPER) Method in Pakistan
Source: Int J Environ Res Public Health. 2022 Apr 21;19(9):5068. doi: 10.3390/ijerph19095068 (PMC9104477; doi:10.3390/ijerph19095068)
Supplement: Supplementary file 1 [file ijerph-19-05068-s001.zip › ijerph-1611886-supplementary.pdf]

Table.S1 Demographic data of study area.
Source: Pakistan Bureau of Statistics

District	Tehsil	Check No	Population
Layyah	Karor Lal-Esan	110/TDA	1,658
Layyah	Karor Lal-Esan	111/TDA	1,398
Layyah	Karor Lal-Esan	116/TDA	1,963
Layyah	Karor Lal-Esan	120/TDA	684
Layyah	Karor Lal-Esan	131/TDA	1,829
Layyah	Karor Lal-Esan	150/TDA	1,780
Layyah	Karor Lal-Esan	152/TDA	1,868
Layyah	Karor Lal-Esan	168/TDA	2,681
Layyah	Karor Lal-Esan	275/TDA	1,764
Layyah	Karor Lal-Esan	279/TDA	3,329
Layyah	Karor Lal-Esan	282/TDA	2,798
Layyah	Karor Lal-Esan	305/TDA	2,513
Layyah	Karor Lal-Esan	316/TDA	1,481
Layyah	Karor Lal-Esan	319/TDA	372
Layyah	Karor Lal-Esan	325/TDA	417
Layyah	Karor Lal-Esan	339/TDA	3,215
Layyah	Karor Lal-Esan	347/TDA	2,458
Layyah	Karor Lal-Esan	348/TDA	1,136
Layyah	Karor Lal-Esan	375/TDA	1,108
Layyah	Karor Lal-Esan	380/TDA	1,347
Layyah	Karor Lal-Esan	407/TDA	749
Layyah	Karor Lal-Esan	419/TDA	1,505
Layyah	Karor Lal-Esan	464/TDA	1,388
Layyah	Karor Lal-Esan	230/TDA	2,017
Layyah	Karor Lal-Esan	230A/TDA	922
Layyah	Karor Lal-Esan	239A/TDA	1,224
Layyah	Karor Lal-Esan	241/TDA	1,428
Layyah	Karor Lal-Esan	242/TDA	618
Layyah	Karor Lal-Esan	243/TDA	1,152
Layyah	Karor Lal-Esan	246/TDA	864

Table.S2 Sample size of the population.

Sample Size before applying exclusion criteria			540
**Samples Excluded**	**Exclusion Criteria**	**Questionnaires excluded**	**Percentage**
	Participants less than the age of 18	15	2.7
	Incomplete response	25	4.6
	Total excluded questionnaires	40	7.4
Sample size after applying exclusion criteria			500
